# CD4^+^ T cells play a crucial role for lenalidomide *in vivo* anti-tumor activity in murine multiple myeloma

**DOI:** 10.18632/oncotarget.5506

**Published:** 2015-10-03

**Authors:** Liang Zhang, Enguang Bi, Sungyoul Hong, Jianfei Qian, Chengyun Zheng, Michael Wang, Qing Yi

**Affiliations:** ^1^ Department of Lymphoma/Myeloma, Division of Cancer Medicine, University of Texas MD Anderson Cancer Center, Houston, TX, United States; ^2^ Deparment of Cancer Biology, Lerner Research Institute, Cleveland Clinic, Cleveland, OH, United States; ^3^ Department of Pharmacy, College of Pharmacy, Seoul National University, Seoul, South Korea; ^4^ Department of Hematology, Second Hospital of Shandong University, Jinan, PR China

**Keywords:** myeloma, 5TGM1, lenalidomide, immunomodulatory, CD4^+^ T cells

## Abstract

Lenalidomide modulates the host immune response against myeloma via multiple actions. Although these effects have been elucidated *in vitro*, the central action of lenalidomide-mediated anti-myeloma immune response *in vivo* is not clear. To investigate its immune action *in vivo*, we selected the murine myeloma cell line 5TGM1, which is resistant to direct tumoricidal effects of lenalidomide *in vitro* and in immunodeficient mice, but sensitive to lenalidomide treatment in 5TGM1-bearing immunocompetent mice. Depletion of CD4^+^ T cells, but not NK cells, B cells, or CD8^+^ T cells, deprived lenalidomide of its therapeutic effects on 5TGM1-bearing immunocompetent mice. Lenalidomide significantly increased the numbers of IFN-γ-secreting CD4^+^ and CD8^+^ T cells but had no effects on NK cells and B cells in this mouse model. Lenalidomide slightly decreased the number of CD25^+^Foxp3^+^ T cells but increased perforin expression in CD8^+^ T cells *in vivo*. Using this mouse model for investigation of anti-tumor immune action of lenalidomide, we demonstrated that lenalidomide facilitated a type-1 anti-tumor immune response *in vivo*. The CD4^+^ T cell subset may play a critical role in the lenalidomide-mediated anti-myeloma immune response *in vivo*.

## INTRODUCTION

Lenalidomide (CC-5013, Revlimid) has been approved by the Food and Drug Administration to treat patients with multiple myeloma because it has potent anti-myeloma activities and a better toxicity profile than thalidomide. Lenalidomide's anti-myeloma mechanisms include tumoricidal, immunomodulatory, anti-inflammatory, and anti-angiogenic properties. The direct tumoricidal effect of lenalidomide occurs through caspase activation and apoptosis induction. [1] It also disrupts the stromal cell-enriched tumor microenvironment and inhibits angiogenesis. [2, 3] Lenalidomide enhances immune cell function not only by activating T and NK cells and increasing their cytokine secretion but also by decreasing IL-6 secretion *in vitro*. [4, 5] Lenalidomide synergistically enhances antibody-dependent cell-mediated cytotoxicity in myeloma cells. [6] All *in vitro* preclinical studies suggest these dual tumoricidal and immunomodulatory activities for lenalidomide. However, the principal action of lenalidomide in the anti-myeloma immune response *in vivo* is unclear.

The 5TGM1 myeloma cell line was initially derived from a murine myeloma cell line, 5T, that originated spontaneously from C57BL/KaLwRij mice [7]. After *i.v.* injection of 5TGM1 cells into C57BL/KaLwRij immunocompetent mice, 5TGM1 myeloma cells thrived and migrated to bone marrow. Similar to myeloma patients, the 5TGM1 myeloma mouse model presented with monoclonal gammopathy and demonstrated marrow replacement, focal osteolytic bone lesions, hind limb paralysis, and occasional hypercalcemia [8].

Our preliminary data showed that 5TGM1 cells were resistant to lenalidomide *in vitro* and in severe combined immunodeficiency (SCID) mice but were sensitive to lenalidomide in an immune response-dependent manner in immunocompetent C57BL/KaLwRij mice *in vivo*. Therefore, 5TGM1-bearing C57BL/KaLwRij mice were taken as an ideal model for investigating the anti-myeloma immune action of lenalidomide *in vivo*. Here, we found that lenalidomide failed to prolong the survival of mice after CD4^+^ T cell but not CD8^+^ T cell, B cell, or NK cell depletion and this was associated with a stronger Th1 response. These results suggest that CD4^+^ T cells are indispensable to a lenalidomide-mediated anti-myeloma immune response *in vivo*.

## RESULTS

### 5TGM1-bearing C57BL/KaLwRij mouse is an ideal model to study the immunomodulatory effect of lenalidomide *in vivo*

**Figure 1:**
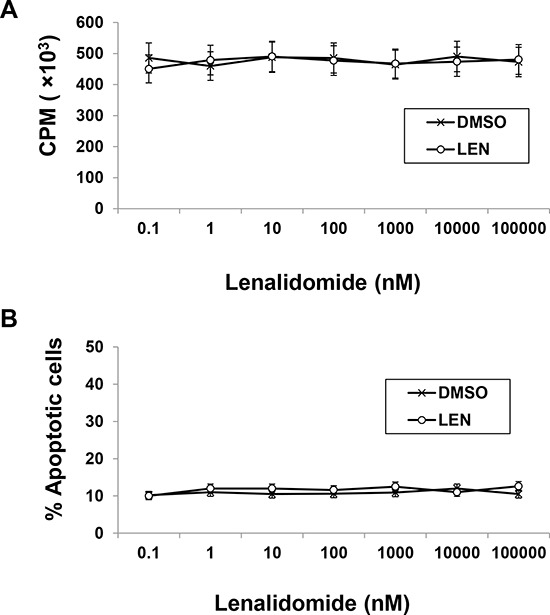
Murine myeloma 5TGM1 cells are resistant to lenalidomide *in vitro* 5TGM1 cells were cultured for 72 hours in the presence of the indicated concentrations of lenalidomide or equal volume of DMSO (vehicle control). **A.** Cell proliferation was assessed by ^3^H-thymidine incorporation assay. **B.** Apoptosis was assessed by annexin V and PI staining. Results of three independent experiments are shown. LEN, lenalidomide; DMSO, dimethyl sulfoxide.

Due to the complicated antitumor activity of lenalidomide, separating its effects into tumoricidal and immunomodulatory activities is difficult, which hampers study of its direct immunomodulatory effects *in vivo*. We wanted a murine myeloma model that is resistant to lenalidomide's tumoricidal effect but sensitive to its immunomodulatory effect. Through *in vitro* treatment with lenalidomide of different myeloma cell lines and analysis of proliferation and apoptosis (data not shown), we decided to focus on 5TGM1 murine myeloma cells. Lenalidomide at concentrations up to 100 μM for 72 hours didn't induce growth inhibition or apoptosis in 5TGM1 myeloma cells (Figure 1).

**Figure 2:**
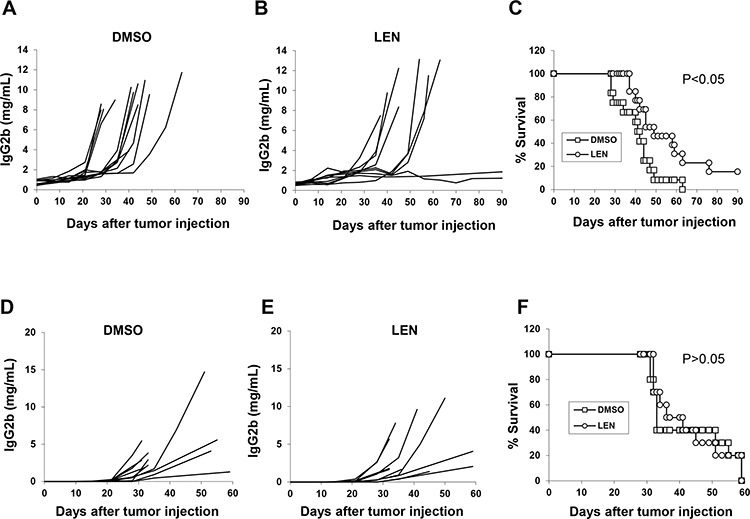
*In vivo* effect of lenalidomide in myeloma-bearing mice C57BL/KaLwRij (**A–C**, 12 mice per group) or B6-SCID (**D–F**, 10 per group) mice were challenged with 2 × 10^6^ 5TGM1 cells via intravenous injection. After 1 week, mice received intraperitoneal injections of lenalidomide (25 mg/kg/day) or equal volume of DMSO for 21 consecutive days. Serum samples were collected weekly, and tumor burden was monitored by measuring circulating IgG2b M-protein. Concentration curves of serum IgG2b M-protein from mice receiving DMSO as vehicle control A and D. or lenalidomide B and E. C and F. Mouse survival curves. LEN, lenalidomide.

A 7-day culture with 100 μM lenalidomide did not affect 5TGM1 growth or apoptosis (data not shown). Because lenalidomide had no direct cytotoxicity on 5TGM1 cells *in vitro*, we next determined whether lenalidomide had any effect on the tumor cells *in vivo*. As shown in Figure 2A–2C, lenalidomide inhibited tumor growth and prolonged survival of 5TGM1-bearing C57BL/KaLwRij mice. Notably, 2/12 mice treated with lenalidomide were tumor-free at post-inoculation day 90 (Figure 2B–2C, *P* < 0.05). However, in immunodeficient B6-SCID mice, which lack T and B cells, lenalidomide treatment failed to inhibit tumor growth (Figure 2D–2E, *P* > 0.05) or prolong survival of tumor-bearing mice (Figure 2F, *P* > 0.05). That lenalidomide had no direct tumoricidal effect on 5TGM1 cells *in vitro* and inhibited myeloma growth in immunocompetent but not immunodeficient mice indicates that the host immune system must play an important role in the anti-myeloma activity of lenalidomide *in vivo* and this activity can be studied in the 5TGM1-bearing C57BL/KaLwRij model.

### NK cells are not the major effector cells for anti-myeloma activity of lenalidomide *in vivo*

**Figure 3:**
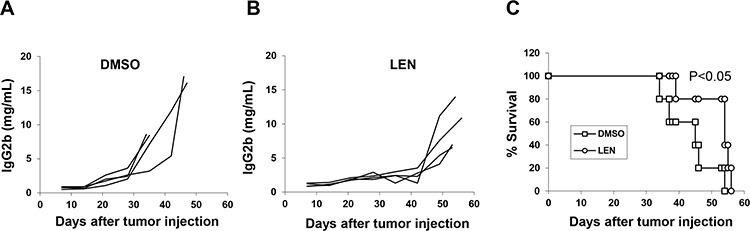
The role of NK cells in the lenalidomide-mediated immunomodulatory effect in 5TGM1-bearing C57BL/KaLwRij mice Mice were treated as described in Figure 2. After receiving 5TGM1 cells, mice were injected with asialo-GM1 antibody twice per week until the end of drug treatment to deplete NK cells. Concentration curve for serum IgG2b M-protein in NK-depleted C57BL/KaLwRij mice receiving DMSO as vehicle control **A.** or lenalidomide **B, C.** Mouse survival curve of NK-depleted C57BL/KaLwRij mice receiving DMSO or lenalidomide (4 mice per group).

Therefore, we first investigated the importance of NK cells in lenalidomide-mediated anti-myeloma activity *in vivo*. Our study in B6-SCID mice showed that lenalidomide had no obvious anti-myeloma effects *in vivo* (Figure 2D–2F). As these SCID mice have functional NK cells but no T and B cells, this result suggested that NK cells may not be important for lenalidomide-mediated anti-myeloma activity *in vivo*. After NK cells were depleted in 5TGM1-bearing C57BL/KaLwRij mice, lenalidomide still retarded tumor growth and prolonged survival (Figure 3, *P* < 0.05). Together with the finding that lenalidomide had an anti-myeloma effect in immunocompetent but not in B6-SCID mice, which have NK cells, these results demonstrated that NK cells are not the main effector cells of lenalidomide action *in vivo*.

### CD4^+^ T cells mediate the pivotal anti-myeloma activity of lenalidomide *in vivo*

**Figure 4:**
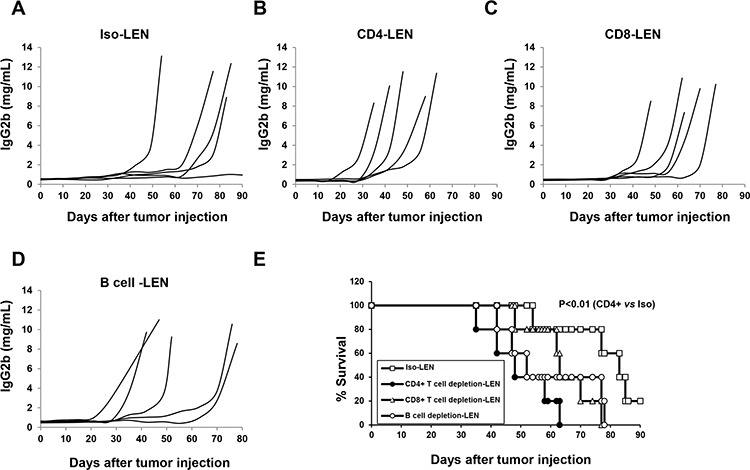
The role of CD4+, CD8+ T and B cells in the lenalidomide-mediated immunomodulatory effect in 5TGM1-bearing C57BL/KaLwRij mice The same procedures as described in Figures 2 and 3 were used for NK cell depletion, lenalidomide treatment and measurement. CD4^+^ T cells, CD8^+^ T cells, and B cells were depleted separately in 5TGM1-bearing C57BL/KaLwRij mice. Concentration curves for serum IgG2b M-protein in 5TGM1-bearing C57BL/KaLwRij mice with isotype IgG **A.** CD4^+^ T cell-depleted 5TGM1-bearing C57BL/KaLwRij mice **B.** CD8^+^ T cell-depleted 5TGM1-bearing C57BL/KaLwRij mice **C.** and B cell-depleted 5TGM1-bearing C57BL/KaLwRij mice **D, E.** Survival curves for CD4^+^ T cell-, CD8^+^ T cell- or B cell-depleted 5TGM1-bearing C57BL/KaLwRij mice versus 5TGM1-bearing C57BL/KaLwRij mice without depletion (5 mice per group).

Next, we investigated whether the lenalidomide immunomodulatory effect depends on T or B cell activity. Depleting CD4^+^T cells significantly enhanced tumor growth and shortened survival (Figure 4A, 4B and 4E; *P* < 0.01, vs. isotype control). Depleting CD8^+^ T cells or B cells did not significantly affect tumor growth or survival (Figure 4A, 4C, 4D and 4E, *P* > 0.05, vs. isotype control). These *in vivo* results demonstrated that CD4^+^ T cells but not CD8^+^ or B cells are crucial in the lenalidomide-mediated anti-myeloma immune response *in vivo*.

### Lenalidomide facilitates a T-cell dependent type I response in 5TGM1-bearing mice

To further understand how lenalidomide affects immune cell function *in vivo*, 5TGM1-bearing mice were sacrificed 1 week after the final lenalidomide injection and the splenocytes were harvested, analyzed directly or restimulated *ex vivo* (see below) before assay.

**Figure 5:**
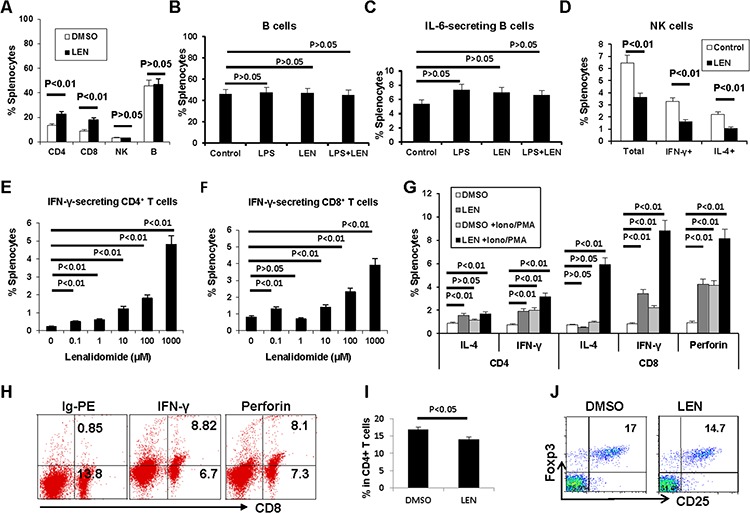
Lenalidomide promotes the expansion of T cells in 5TGM1-bearing C57BL/KaLwRij mice Splenocytes from myeloma-bearing C57BL/KaLwRij mice were analyzed directly (A) or restimulated *ex vivo* for 72 hours (B–J) Percentages of **A.** CD4^+^ T cells, CD8^+^ T cells, NK cells, and B cells, **B-C.** B cells and IL-6 secreting B cells, **D.** NK cells including IFN-γ-secreting and IL-4-secreting NK cells, **E.** IFN-γ-secreting CD4^+^ T cells, and **F.** IFN-γ-secreting CD8^+^ T cells. **G.** The synergistic effect of lenalidomide with PMA/ionomycin on the activation of CD4^+^ T cells and CD8^+^ T cells. **H.** Representative flow cytometry results showing CD8^+^ T cell activation. **I.** Percentage of CD25^+^ Foxp3^+^ T cells after restimulation with lenalidomide. **J.** Representative flow cytometry results for CD25^+^ Foxp3^+^ T cells. PMA, phorbol 12-myristate 13-acetate; Ion, ionomycin; LPS, lipopolysaccharide; Ig-PE, R-phycoerythrin-conjugated rat immunoglobulin.

First the percentages of splenic CD4^+^ T cells, CD8^+^ T cells, NK cells, and B cells were analyzed by flow cytometry. As Figure 5A shows, the percentages of both CD4^+^ T cells and CD8^+^ T cells increased about 2-fold vs. vehicle control (*P* < 0.01). NK cells and B cells showed no change (*P* > 0.05).

After restimulation of splenocytes with lenalidomide for 72 hours *ex vivo*, the B cell population showed no significant change even when treatment was combined with LPS (Figure 5B–5C; *P* > 0.05). The total number of NK cells, including IFN-γ-secreting and IL-4-secreting NK cells, decreased (Figure 5D; *P* < 0.01).

We found that lenalidomide significantly increased IFN-γ secretion in both CD4^+^ and CD8^+^ T cells in 5TGM1-bearing mice (Figure 5E–5F). When lenalidomide was combined with PMA/ionomycin, synergistic effects on IFN-γ production by CD4^+^ and CD8^+^ T cells and expression of perforin by CD8^+^ T cells were observed (Figure 5G–5H). Interestingly, lenalidomide and PMA/ionomycin also stimulated CD8^+^ but not CD4^+^ T cells to secrete significantly more IL-4 (Figure 5G). Lenalidomide alone slightly decreased the percentage of CD25^+^Foxp3^+^CD4^+^ regulatory T (Treg) cells (Figure 5I). Taken together, these data clearly showed that lenalidomide enhances the type I anti-myeloma CD4^+^ and CD8^+^ T cell responses *in vivo*.

## DISCUSSION

Lenalidomide, a second-generation immunomodulatory drug, exhibits antitumor activity. Lenalidomide-containing therapeutic regimens resulted in higher response and improved outcomes in the patients with MM relapse or with refractory MM [10, 11]. Previous studies demonstrated that lenalidomide had a direct tumoricidal role by inducing apoptosis and blocking tumor cell proliferation [12, 13]. Recent *in vitro* studies showed that lenalidomide bound to cereblon and then selectively degraded specific transcription factors — the Ikaros family zinc finger proteins 1 and 3 (IKZF1 and IKZF3) — which were necessary for myeloma survival [14, 15]. However, it is essential to investigate how lenalidomide modulates the immune response *in vivo*. Considering the complicated effects of lenalidomide on tumor cells and immune cells, the mechanistic study of immunomodulation is limited by the availability of a suitable tumor model that can separate these two effects. In the current study, we found that a murine myeloma cell line, 5TGM1, was totally resistant to lenalidomide toxicity *in vitro*. Most importantly, we found that lenalidomide inhibited tumor growth and prolonged the survival of 5TGM1-bearing C57BL/KaLwRij mice; however, it failed to inhibit tumor growth of 5TGM1-bearing SCID mice. Those results demonstrated that lenalidomide affect 5TGM1-bearing mice survival in an immune response-dependent manner only. This 5TGM1 myeloma model facilitates study of the immunomodulatory role without the direct tumoricidal effect of lenalidomide *in vivo*.

By depleting different cell populations in the 5TGM1-bearing immunocompetent mice separately, we found that CD4^+^ T cells play a critical role in the lenalidomide-mediated antitumor immune response. Under different types of stimulation, CD4^+^ T cells will differentiate into different populations, including Th1, Th2, Th9, Th17, TfH, and regulatory T cells, to mediate different immune responses. Lenalidomide potentiated T cell activation in a costimulatory manner *in vitro* [16]. IL-2 is T cell activation hallmark and its transcription was suppressed by IKZF3, which bound to the IL-2 promoter directly [17]. Lenalidomide promoted IKZF3 degradation, thus releasing the suppressive effect of IKZF3 on IL-2 transcription and promoting T cell activation [14, 15].

Our data showed that lenalidomide enhanced IFN-γ production in CD4^+^ T cell *in vitro* and *in vivo*, suggesting an increased Th1 response after lenalidomide treatment. Th1 polarized immune responses are associated with CD8^+^ T cell-mediated cytotoxicity against tumor cells and induce tumor regression. After lenalidomide treatment, CD8^+^ T cells produced more IFN-γ and perforin, which is consistent with the enhanced Th1 response. The cytotoxic effect of CD8^+^ T cells can be inhibited through recruitment and/or conversion of natural or adaptive Tregs. Our data showed that the number of Tregs was somewhat decreased in tumor-bearing C57BL/KaLwRij mice after lenalidomide treatment. An *in vitro* study by Luptakova et al demonstrated that lenalidomide resulted in T cell polarization toward a Th1 phenotype characterized by increased IFN-γ secretion of T cells while decreasing the number of Tregs [18], which is consistent with our *in vivo* data.

The exact molecular mechanism by which lenalidomide regulates CD4^+^ T cell activation and polarization is not clear. In the absence of CD28 stimulation, lenalidomide induced IL-2, IFN-γand TNF-α secretion [19], suggesting that lenalidomide may activate T cells by mimicking the costimulatory signaling. Furthermore, LeBlance et al found that lenalidomide increased tyrosine phosphorylation in the CD28 receptor intracellular domain in the absence of costimulatory molecules [20].

Although cereblon is essential for lenalidomide-mediated direct tumoricidal activity against myeloma, it also mediated T cell activation by lenalidomide or its analogues because knockdown of cereblon in primary human T cells abrogates drug-induced IL-2 expression [21]. Furthermore, lenalidomide promoted the cereblon-dependent destruction of Ikaros proteins [15]. IKZF1 (Ikaros) directly bound to the promoter region of T-bet, the master regulator of Th1 cell differentiation, and suppressed IFN-γ production and promoted Th2 polarization [22]. IKZF2 (Helios) directly bound to Foxp3 promoter and up-regulated Foxp3 expression, which results Treg differentiation [23]. IL-2 transcription was suppressed by IKZF3 through binding of its promoter. Thus, although it is not known whether lenalidomide can degrade IZKF2 as well as IZKF1 and IZKF3, it seems that lenalidomide can promote T cell activation by down-regulation of IZKF1, polarize Th1 response by degradation of IZKF3, and may slightly decrease Treg by down-regulation of the Ikaros family.

Previous studies showed that lenalidomide activated NK cells and enhanced NK cell function in MM patients, and its effects on NK cells were dependent on CD4^+^T cells and IL-2 secretion [24, 25]. Our results demonstrated that NK cells were not a therapeutic target of lenalidomide. Therefore, lenalidomide may affect NK cell function in the presence of CD4^+^ T cells.

Our study demonstrates that CD4^+^ T cells are critical to the lenalidomide-mediated antitumor immune response *in vivo* and the number of CD4^+^ T cells was significantly increased. Therefore it is necessary to analyze whether there is a positive correlation between the number of CD4^+^ T cells and response to lenalidomide in myeloma patients.

## MATERIALS AND METHODS

### Mice and cell lines

Male C57BL/KaLwRij mice were purchased from Harlan, and CPB.B6.CB17-Prkdc^SCID^/Szj (B6 SCID) mice were purchased from Jackson Laboratory. The Institutional Animal Care and Use Committee of the University of Texas M.D. Anderson Cancer Center approved the study. The 5TGM1 myeloma cell line was derived from 5T murine myeloma that spontaneously arose in C57BL/KaLwRij mice and was cultured in Iscove's modified Dulbecco's medium (Invitrogen) supplemented with 10% heat-inactivated fetal bovine serum (Atlanta Biologicals), 100 units/mL penicillin-streptomycin, and 2 mmol/L L-glutamine (Invitrogen).

### Drugs and reagents

Lenalidomide was provided by Celgene (Summit, NJ) and dissolved in dimethyl sulfoxide (DMSO) at a stock concentration of 0.5 M. FITC-conjugated annexin V was purchased from Caltag Laboratories (Burlingame, CA). Propidium iodide (PI), lipopolysaccharide (LPS), ionomycin, and phorbol 12-myristate 13-acetate (PMA) were purchased from Sigma-Aldrich (St. Louis, MO). Fetal bovine serum was purchased from Atlanta Biologicals (Norcross, CA). ^3^H-thymidine was purchased from Amersham (Arlington Heights, IL).

### *In vivo* therapeutic effect of lenalidomide

Mice were challenged intravenously with 2 × 10^6^ 5TGM1 myeloma cells. Three weeks later, when myeloma growth was established, mice received intraperitoneal lenalidomide (25 mg/kg) or equal volume vehicle control daily for 21 consecutive days. Tumor burden was monitored by measuring serum IgG2b M-protein by ELISA. Mice were euthanized when moribund or when hind-leg paralysis developed.

### Cell proliferation assay

The inhibitory growth effect of lenalidomide on myeloma cells was assessed by ^3^H-thymidine incorporation assay. Briefly, cells were plated in 96-well plates at a density of 5 × 10^4^ cells/well and treated with different concentrations of lenalidomide for 72 hours. ^3^H-thymidine, 1 μCi, was added to each well, and the cells were incubated for an additional 16 hours. The cells were washed, and the radioactivity was measured using a scintillation beta-counter (PerkinElmer Life and Analytical Sciences, Shelton, CT). The data are expressed as the percentage of the DMSO control value.

### Apoptosis assay

An annexin V-binding assay was used to detect the induction of apoptosis. Cells were seeded in 48-well plates with different concentrations of lenalidomide for 72 hours. To quantify the percentage of cells undergoing apoptosis, cells were washed twice with cold phosphate-buffered saline (PBS) and then resuspended in 1X binding buffer at 1 × 10^6^ cells/mL. Subsequently, 100 μl cell suspension solution was transferred into a 5-mL tube and 5 μl annexin V and PI were added. The tubes were gently vortexed and incubated for 15 minutes at room temperature in the dark. Then 300 μl 1X binding buffer was added and analyzed immediately using a FACScan flow cytometer (Becton Dickinson, San Jose, CA). The number of apoptotic cells was determined by counting annexin V-positive cells.

### Enzyme-linked immunosorbent assay (ELISA)

5TGM1 myeloma cells secrete mouse immunoglobulin IgG2b Id protein. The level of mouse circulating IgG2b Id protein was used to monitoring tumor burden. As described previously [9], mouse serum was collected at the indicated times, and the amounts of secreted IgG2b Id protein in mouse serum were quantified by ELISA.

### Flow cytometry

An aliquot of freshly isolated cells from the spleen of lenalidomide/DMSO treated C57BL/KaLwRij mice was incubated with fluorescent isothiocyanate (FITC)-, phycoerythrin (PE)-, or allophycocyanin (APC)-conjugated monoclonal antibodies (mAbs) against CD4, CD8, B220, or NK1.1 (eBioscience, San Diego, CA) for 30 minutes at 4°C, and then washed twice and analyzed by flow cytometry. Another aliquot of freshly isolated cells was incubated with 1 μM lenalidomide or DMSO (vehicle control) with and without LPS (100ng/mL) or ionomycin (1 μg/mL) plus PMA (300 ng/mL) at 37°C for 72 hours. These cells were then subjected to CD4, CD8, B220, or NK1.1 cell surface staining, and then treated using a Cytofix/Cytoperm kit (BD Biosciences) for intracellular staining for IL-4, IFN-γ, perforin or Foxp3 (eBioscience, San Diego, CA).

### *In vivo* T cell, B cell, and NK cell depletion

Hybridomas producing anti-CD4 mAb GK1.5 (rat IgG2b) and anti-CD8 mAb 2.43 (rat IgG2b) were obtained from the American Type Culture Collection to deplete CD4 and CD8 cells. Antibodies were purified using HiTrap Protein D columns (GE Healthcare). Anti-mouse B220 mAb was purchased from R&D Systems to deplete B cells. Anti-mouse asialo-ganglio N-tetraoglyceramide (asislo-GM1) polyclonal antibody (Cedarlane, Canada) was used to deplete NK cells. Cell depletion was performed by intraperitoneal injection of 100 μg of antibody against CD8, CD4, B220, or asislo-GM1 twice per week before and during treatment. The efficiency of cell depletion was assessed by staining peripheral blood mononuclear cells with CD4-PE, CD8-FITC, B220-PE, or NK1.1-FITC (eBioscience).

### Statistical analysis

All assays were performed in triplicate and expressed as mean ± SE. Statistical significance was determined by Student's *t* test. Mouse overall survival was determined using the Kaplan-Meier method. *P* values < 0.05 were considered significant.
